# Current Review of Iron Overload and Related Complications in Hematopoietic Stem Cell Transplantation

**DOI:** 10.4274/tjh.2016.0450

**Published:** 2017-03-01

**Authors:** Erden Atilla, Selami K. Toprak, Taner Demirer

**Affiliations:** 1 Ankara University Faculty of Medicine, Department of Hematology, Ankara, Turkey

**Keywords:** iron overload, Hematopoietic stem cell transplantation, ferritin, Iron chelation

## Abstract

Iron overload is an adverse prognostic factor for patients undergoing hematopoietic stem cell transplantation (HSCT). In the HSCT setting, pretransplant and early posttransplant ferritin and transferrin saturation were found to be highly elevated due to high transfusion requirements. In addition to that, post-HSCT iron overload was shown to be related to infections, hepatic sinusoidal obstruction syndrome, mucositis, liver dysfunction, and acute graft-versus-host disease. Hyperferritinemia causes decreased survival rates in both pre- and posttransplant settings. Serum ferritin levels, magnetic resonance imaging, and liver biopsy are diagnostic tools for iron overload. Organ dysfunction due to iron overload may cause high mortality rates and therefore sufficient iron chelation therapy is recommended in this setting. In this review the management of iron overload in adult HSCT is discussed.

## Abstract (tr)

Demir yüklenmesi, hematopoietik kök hücre nakli (HKHN) yapılan hastalarda görülen olumsuz prognostik göstergedir. Pretransplant ve erken posttransplant ferritin ve transferrin satürasyonlarının yüksekliği, transfüzyon ihtiyacına bağlıdır. Posttransplant demir yüklenmesi; enfeksiyonlar, hepatik sinüzoidal obstrüksiyon sendromu, mukozit, karaciğer disfonksiyonu ve akut graft versus host hastalığı ile ilişkili olarak bulunmuştur. Hiperferritinemi, pre ve posttransplant sağkalım oranlarında düşüklüğe neden olur. Demir yüklenmesinin tanısında serum ferritin düzeyleri, magnetik rezonans görüntüleme ve karaciğer biyopsisi kullanılır. Demir yüklenmesine bağlı organ disfonksiyonu yüksek mortalite oranlarıyla ilişkilidir ve bu durumda yeterli demir şelasyon tedavisi önerilmektedir. Bu derlemede erişkin HKHN’de demir yüklenmesine yaklaşım tartışılmıştır.

## INTRODUCTION

Hematopoietic stem cell transplantation (HSCT) is an established treatment approach in a variety of hematological disorders but is still complicated with excessive mortality and morbidity despite advances in conditioning regimens and infectious disease management [1,2,3,4,5]. Today high-dose therapy and auto-HSCT is a treatment option in selected hematopoietic and nonhematopoietic tumors [4]. The common early complications include infections and mucositis [5]. Allo-HSCT is recommended in congenital or acquired bone marrow failures and hematological malignancies. Sinusoidal obstruction syndrome (SOS), hemorrhagic cystitis, engraftment failure, idiopathic pneumonia syndrome, infection, and graft-versus-host disease (GVHD) are major causes of morbidity and non-relapse mortality (NRM) [6]. Late complications of HSCT mainly involve skin, oral mucosa, ocular, gastrointestinal, pulmonary, endocrine, metabolic, infectious, renal, neurological, psychosocial, and cardiovascular systems, as well as secondary malignancies [6,7].

Iron overload is a common condition in patients with hematological malignancies and HSCT recipients. The incidence of iron overload in auto-HSCT is around 34%, less frequent than in allo-HSCT [8]. In the allo-HSCT setting, the incidence of iron overload varies between 30% and 60% [9,10]. Sucak et al. retrospectively investigated 24 liver biopsies for evaluation of the cause of liver dysfunction after allo-HSCT. Iron overload was detected in a total of 75% of these liver biopsy samples and as a sole histopathologic abnormality in 33% of recipients [11]. The main factor in the high incidence of iron overload in both transplants is exposure to red blood cell (RBC) transfusions both during initial treatment and in the posttransplant period [10].

This review will focus on normal iron hemostasis and mechanisms of iron overload in HSCT recipients and the effects and management of excess iron in the setting of HSCT.

## IRON HOMEOSTASIS AND THE MECHANISMS OF IRON OVERLOAD

Iron is an essential element for many enzymatic functions and hemoglobin synthesis. There are four major cell types determining the iron content and distribution: duodenal enterocytes, erythroid precursors, reticuloendothelial macrophages, and hepatocytes. The iron cycle in the body starts with duodenal enterocyte absorption of 1 to 2 mg of iron per day. Iron binds to transferrin and is taken up by erythroid precursors for heme synthesis. Reticuloendothelial macrophages clear erythrocytes and release the iron from heme in order to export it to the circulation and store it in the form of ferritin. Hepatocytes are the major cells for iron storage as ferritin and the production of the peptide hormone hepcidin. However, in the state of an excess of iron, reactive oxygen species (ROS) affect the functions of organs such as the liver, heart, and endocrine glands [12]. In patients receiving regular transfusions, tissue iron deposition can begin within 1-2 years; however, clinically evident cardiac or hepatic dysfunction may not occur for 10 years or more [10]. Excess iron is also associated with the prooxidant effects that contribute to DNA damage and the promotion of oncogenesis.

There are many ongoing studies related to erythroid regulators of iron homeostasis. Hepcidin is the main regulator of iron absorption and tissue distribution that controls iron in the plasma by absorption of dietary iron in the intestines, recycling of iron by macrophages, and mobilization from hepatocyte storage. Hepcidin promotes the degradation of ferroportin, leading to retention of iron in iron-exporting cells and decreased flow of iron into the plasma [13]. In inherited anemias with ineffective erythropoiesis, beta-thalassemia, and congenital dyserythropoietic anemia, pathological suppression of hepcidin synthesis and hyperabsorption of dietary iron occurs [14]. In thalassemia, twisted-gastrulation 1 was proposed as pathological suppressors of hepcidin [15]; however, its role was not defined. Kautz et al. [16] reported a new erythroid regulator, which is essential for early suppression of hepcidin after erythropoietic stimulation and named “erythroferrone” (ERFE). If it is confirmed in clinical studies, ERFE neutralization could be a new treatment strategy in iron overload in iron-loading anemias.

Several clinical reports showed that iron chelation therapy improved hematopoiesis in iron-overloaded patients with myelodysplastic syndrome (MDS) [17,18]. Recently, for investigating the impact of iron deposition on hematopoiesis, researchers initiated studies in vivo. Okabe et al. examined iron-overloaded mice and hematopoietic parameters as well as the bone marrow microenvironment. They showed that hematopoietic parameters of the peripheral blood did not change; however, myeloid progenitor cells in the bone marrow were increased. The number and the function of erythroid progenitors remained the same. Bone marrow transplantation to iron-overloaded mice resulted in delayed hematopoietic reconstitution. The levels of erythropoietin and thrombopoietin were significantly low in iron-overloaded mice compared to the normal group. The authors concluded that excess iron disrupts the hematopoietic microenvironment [19]. Zhang et al. evaluated the effect of iron overload on the bone marrow microenvironment in mice and found that chemokine stromal cell-derived factor-1, stem cell factor-1, and vascular endothelial growth factor-1 expressions were decreased. The decreased hematopoietic functions were influenced by elevated phosphatidylinositol 3 kinase and reduced forkhead box protein mRNA expression, which could induce generation of ROS. These data showed that iron overload could impair the bone marrow microenvironment [20]. Chai et al. showed that iron overload markedly decreased the ratio and clonogenic function of murine hematopoietic stem and progenitor cells by elevation of ROS [21].

## IRON OVERLOAD AND RELATED COMPLICATIONS IN HEMATOPOIETIC STEM CELL TRANSPLANTATION

Iron overload is a prominent problem in HSCT recipients. HSCT recipients receive large RBC transfusions both during the pre- and peritransplant periods. In addition to that prolonged dyserythropoiesis, increased intestinal iron absorption due to chemotherapy-associated mucositis and release of iron from damaged tissues raise iron to undesired levels [10]. Chemotherapy and radiotherapy-associated hepatic damage may also contribute to the release of iron stores and diminish transferrin synthesis [22,23]. In an autologous HSCT mice model, iron overload was detected to be associated with increased melphalan and busulfan toxicities through a pharmacodynamic interaction [24]. In a recent study, the interacting effects of total body irradiation and cell transplantation on the expression of iron regulatory genes had contributed to iron overload in murine recipients [25].

Armand et al. [26] retrospectively analyzed the impact of elevated pretransplant serum ferritin levels in 590 patients undergoing myeloablative stem cell transplantation. In that analysis, a strong relationship was detected between pretransplant ferritin levels and survival rates. The 5-year overall survival (OS) for patients with pretransplant ferritin levels in the first quartile (0-231 ng/mL) was 54% (95% confidence interval [CI], 45%-63%); in the second quartile (232-930 ng/mL), it was 50% (95% CI, 41%-59%); in the third quartile (931-2034 ng/mL), it was 37% (95% CI, 27%-46%); and in the fourth quartile (>2034 ng/mL), it was 27% (95% CI, 18%-36%) (p<0.001). The 5-year disease-free survival rates, from the lowest to highest quartile, were 43% (95% CI, 33%-53%), 44% (95% CI, 35%-54%), 34% (95% CI, 24%-43%), and 27% (95% CI, 19%-36%) (p<0.001). The majority of patients diagnosed with MDS and acute leukemia had an increased risk of mortality (hazard ratio [HR], 2.6, p=0.003; HR, 1.6, p=0.031). The authors also stated that pretransplant ferritin levels in the top quartile were associated with a borderline increase in the risk of veno-occlusive disease (odds ratio [OR], 1.7; 95% CI, 1.0-2.9; p=0.054) [26]. Barba et al. studied the effect of hyperferritinemia in allo-HSCT with reduced intensity conditioning in 201 adult lymphoma patients. In the multivariate analysis, patients with hyperferritinemia at transplantation (>399 ng/mL) showed a lower 4-year OS (HR, 1.8; CI, 1.2-2.8; p=0.008) and higher NRM (HR, 1.8; CI, 1.1-3.2; p=0.03) than those without hyperferritinemia [27]. Mahindra et al. studied hyperferritinemia in an autologous HSCT setting in 315 patients with Hodgkin or non-Hodgkin lymphoma. In multivariate analysis, a pretransplant ferritin level of >685 ng/mL was associated with significantly lower OS (p=0.002) and relapse-free survival (p=0.021) but increased risk of relapse (p=0.005) and relapse-related mortality (p<0.001) [28].

In a metaanalysis, pre-HSCT iron overload was related to poor OS and higher incidence of NRM [29]. Nakamae et al. showed a significant relation of serum ferritin levels at day 30 and 1 year after HSCT with OS [30].

The prognostic impact of iron overload in the posttransplantation period was determined by Meyer et al. in 290 patients who received myeloablative unmanipulated allo-HSCT. Ferritin and transferrin saturation were elevated before and increased in the first months after transplantation as a result of high transfusion needs. Plasma iron levels were found to be variable depending on food intake and time of day. After a peak in the first 1 to 3 months after transplantation, ferritin levels decreased gradually. Hyperferritinemia had a negative effect on survival in all periods (0 to 6 months, p<0.001; 6 to 12 months, p<0.001; 1 to 2 years, p=0.02; 2 to 5 years, p=0.002) and no relation with RBC transfusion dependency or GVHD [31]. On the other hand, Armand et al. evaluated the effect of serum iron parameters as well as liver and cardiac iron deposition by magnetic resonance imaging (MRI) prospectively in 45 patients receiving myeloablative allo-HSCT. They found no significant increase in ferritin levels and liver or cardiac iron content in the 12 months following allo-HSCT. Pretransplant ferritin (as reflected in liver iron content) was not found related to increased mortality, relapse, or GVHD. The authors concluded that prospective studies using direct measurement of iron overload rather than ferritin should be designed [32].

Post-HSCT iron overload was shown to be associated with infections, hepatic SOS, mucositis, liver dysfunction, and acute GVHD [33,34,35,36,37]. Early and late complications of HSCT that have been associated with iron overload are summarized in Table 1. Iron accumulation may cause increased growth and virulence of *Aspergillosis* species [38]. Maertens et al. showed an association of iron overload with mucormycosis in 5 allo-HSCT recipients [39]. Sivgin et al. suggested that higher ferritin levels generally above 1550 ng/mL were associated with invasive fungal pneumonia (IFP) in pretransplant allo-HSCT recipients. Patients with IFP had lower Karnofsky performance status (p<0.05) and poorer OS (39.6 vs. 60.9 months, p=0.015) [40]. Increased risk of hepatosplenic candidiasis was also detected in patients with higher pretransplant ferritin levels [41]. Several other bacterial infections were also detected in iron-overloaded HSCT recipients [10].

Liver dysfunction was evaluated in the allo-HSCT setting with pre- and posttransplant liver biopsies in 25 recipients. Fatal veno-occlusive disease occurred in 2 and biochemical abnormalities in 24 patients. Iron overload was detected increased in posttransplant biopsies (96%, p<0.01) [42]. It was suggested that iron-induced hepatotoxicity is multifactorial and consists of oxidative stress and modulation of gene expression of Kupffer cells [43]. Iron-generated oxyradicals and peroxidation of lipid membranes may also cause cellular injury [44]. It is well known that SOS, which is an important cause of transplant-related mortality of up to 50%, is characterized by the presence of at least 2 of the following features: hyperbilirubinemia, painful hepatomegaly, and weight gain [45]. SOS was diagnosed in 88 patients (21%) at a median of 10 days (range, 2-29 days) in 427 HSCT recipients. Pretransplant serum ferritin level higher than 1000 ng/dL (OR, 1.78; 95% CI, 1.02-3.08) was found to be a risk factor for SOS [46]. This finding was also confirmed by a prospective cohort study of 180 patients receiving HSCT by Morado et al. [34].

Data for determining the role of iron overload in the pathogenesis of GVHD are conflicting and should be confirmed by further studies. Pullarkat et al. evaluated the effect of pretransplant ferritin levels on acute GVHD in a prospective cohort study of 190 allo-HSCT patients. Acute GVHD was more common in patients with high ferritin levels (>1000 ng/mL). The initiating event of pathogenesis was defined as the antigen exposition following increased ROS-mediated tissue injury [35]. However, Mahindra et al. demonstrated the decreased incidence of chronic GVHD associated with pretransplant ferritin levels of >1910 µg/L in 222 patients who underwent myeloablative allo-HSCT [47]. In another study of 264 patients with allo-HSCT, there was no relation detected between serum ferritin levels and acute/chronic GVHD [46]. In fact, elevated pretransplant ferritin levels of >400 µg/L were associated with a lower risk of chronic GVHD (HR, 0.51; 95% CI, 0.33-0.79; p=0.003) in 309 allo-HSCT recipients. The authors hypothesized that ferritin might show an immunosuppressive effect and thus reduce the incidence of GVHD following HSCT [48].

It should be kept in mind that, although advances in supportive care and techniques have improved the survival of HSCT recipients [49,50,51,52], iron overload is still a challenging issue and may be associated with liver fibrosis, heart failure, hypogonadism, diabetes, and an endocrinopathy known as “bronze diabetes” in HSCT recipients as long-term complications [53].

## DIAGNOSIS OF IRON OVERLOAD

The European Group for Blood and Marrow Transplantation, Center for International Blood and Marrow Transplant Research, and American Society of Blood and Marrow Transplantation (ASBMT) guidelines promoted screening of serum ferritin levels in the post-HSCT period for determining the risk of iron overload [54]. In the 2012 ASBMT guidelines, ferritin measurement is recommended in patients who received transfusions in the pre- and posttransplant settings. Generally, the threshold for serum ferritin level is accepted as 1000 µg/L for detection of iron overload [55]. It is recommended in these guidelines that patients with high liver function tests, high transfusion needs, or hepatitis C infection should be monitored subsequently until ferritin levels are below 500 ng/mL [53].

Ferritin level continues to be the mainstay for the clinical evaluation of iron overload and macrophages and T cells are the main sources of ferritin. Both over transfusion and inflammatory reactions may accompany high ferritin levels. In addition to inflammation, ineffective erythropoiesis and liver disease can also be associated with high ferritin levels [13,56]. Researchers hypothesized whether highly increased ferritin concentrations might be related to GVHD-associated inflammation in pediatric patients, but they concluded that ferritin could not be a biomarker of chronic or acute GVHD [57]. In fact, serum ferritin levels appeared to have a poor correlation with liver iron concentration (LIC) in pediatric patients with thalassemia and sickle cell disease [58]. There was a modest correlation (p=0.47) detected by Majhail et al. between serum ferritin and LIC by MRI in allo-HSCT recipients. They indicated that ferritin can be a good screening test but a poor predictor of tissue iron overload and they recommended estimation of LIC before initiating chelation therapy [9]. It was reported that ferritin, in combination with transferrin saturation, has superior prognostic value in determining iron overload when compared to ferritin alone [53].

An alternative marker for determining iron overload is nontransferrin-bound iron (NTBI), which is a low-molecular-weight form of iron. NTBI is formed when transferrin becomes saturated and unable to bind excess iron [59]. There are studies conducted that showed that the level of NTBI was significantly increased in iron overload and might be used to assess the efficacy of chelation in patients with beta-thalassemia major [60]. However, Goto et al. studied the prevalence of iron overload in adult allo-HSCT patients by serum ferritin and NTBI and stated that ferritin was well correlated with NTBI but NTBI was found to be a weaker marker than ferritin in terms of iron overload outcomes. The major issue for this finding was that NTBI only refers to iron in the plasma binding to ligands other than transferrin. Ferritin was confirmed to be correlated with the number of packed RBCs received in patients without active infection, relapse, or second malignancy [61].

Liver biopsy is the gold standard in evaluating iron overload. LIC exceeding 80 µmol/g of liver dry weight is consistent with iron overload with a hepatic index greater than 1.9 mmol/kg/year [55]. The hepatic iron index is the ratio of hepatic iron concentration to the age of the patient in years. Even though liver biopsy can exclude an alternative diagnosis of hepatic dysfunction such as GVHD and infections, the use is limited in HSCT patients because the procedure is invasive and patients usually have low platelet counts.

LIC measurement by MRI has gained importance since it is noninvasive, rapid, and widely available. Today MRI techniques T2 and R2 are reported to have sensitivity and specificity of 89% and 80% in determination of LIC, respectively [62,63]. Ferritin levels of more than 1000 ng/mL were found to be correlated with LIC of >7 mg/g in HSCT survivors [10].

The superconducting quantum interference device (SQUID) can assess total body iron with biomagnetic susceptometry by detecting the paramagnetic materials ferritin and hemosiderin. Although it is the reference standard for estimation of LIC, the technique is complex, expensive, and very limited [64]. Busca et al. showed that LIC measurements obtained by SQUID in the presence of moderate (LIC 1000-2000 µg Fe/g wet weight) or severe (LIC >2000 µg Fe/g wet weight) iron overload were associated with high ferritin levels in 69% of patients [62]. Commonly used diagnostic methods for determining iron overload are summarized in Table 2 [10].

## MANAGEMENT OF IRON OVERLOAD

There is no consensus in the literature on when or how to treat iron overload in HSCT settings. Management of iron overload should be individualized based on several factors such as the need for ongoing RBC transfusion therapy, ability to tolerate iron-depleting therapy, cost-effectiveness, or urgency to reduce body iron stores. Therapy may not be needed in mild cases of iron overload; avoidance of alcohol and iron supplements can be recommended [65]. Phlebotomy and iron chelation agents are two treatment approaches for protecting recipients from long-term end-organ toxicities. As a recommendation, patients with LIC of >15 mg/g dry weight should be treated aggressively with both phlebotomy and chelation; when LIC is 7-15 mg/g dry weight, phlebotomy is indicated; and when LIC is under 7 mg/g dry weight treatment is only indicated if there is evidence of liver disease [53].

In adult survivors of allo-HSCT, unlike large pediatric cohorts, case series were reported regarding the safety and feasibility of phlebotomy [63,64]. In a routine phlebotomy program, approximately 250 mg of iron is removed once or twice weekly [54]. Although phlebotomy has the advantage of better compliance, fewer side effects, and lower costs, the efficacy is limited [53]. Phlebotomy did not have a statistically significant effect on the reduction of ferritin levels before chelation treatment compared with ferritin levels after chelation treatment in a small cohort of patients after allo-HSCT [66]. Phlebotomies were repeated every 1-2 weeks until a serum ferritin level of <500 ng/mL in post-HSCT patients and LIC was significantly reduced in a small cohort (median, 1419 µg Fe/g wet weight to 625 µg Fe/g wet weight; p<0.001) [62]. After normalization of transaminases and serum ferritin levels, maintenance phlebotomy is recommended every 3-6 months to prevent reaccumulation [53].

Deferoxamine is an iron-chelating agent available in vials for intramuscular, subcutaneous, and intravenous administration. It chelates iron from ferritin and hemosiderin, but not readily from transferrin. The common adverse events are reported as localized irritation, pain, burning, swelling at the injection site, and systemic allergic reaction [67]. Deferoxamine has proven efficacy and safety in HSCT recipients with a recommended schedule of at least 5 nights delivered by subcutaneous pump for 8-12 h [64]. Neurotoxicity, ocular toxicity, ototoxicity, and growth retardation have been related to overuse [55]. However, parenteral administration is uncomfortable and time-consuming and it increases the risk of infection; therefore, oral iron chelators have been under investigation. Deferiprone is an oral iron chelator but it has not been investigated in HSCT recipients and is not commercially available in all countries [10].

Deferasirox is an oral iron chelator that was approved by the US Food and Drug Administration in 2005 and improved outcomes in iron overload. The effective dose of deferasirox is between 20 and 40 mg/kg (water soluble tablet: 500 mg). Common side effects include skin rash, nausea, vomiting, diarrhea, and elevation of renal function test results [68]. Deferasirox treatment at a dose of 20 mg/kg/day in hyperferritinemia (ferritin of ≥1000 ng/mL) was analyzed retrospectively in 23 posttransplant patients. Iron (p=0.003), total iron-binding capacity (p=0.025), ferritin (p=0.001), alanine transaminase (p=0.019), and total bilirubin levels (p=0.001) were significantly decreased after treatment. Eight patients (34.7%) who had hemoglobin levels of >12 g/dL also underwent phlebotomy. The reductions of ferritin levels were significant between the deferasirox + phlebotomy group compared to the deferasirox + nonphlebotomy group (p=0.025). The most common adverse effects were nausea and vomiting in 13% of patients while no renal dysfunction was observed. The authors concluded that oral deferasirox treatment was safe and effective with or without phlebotomy in the posttransplant setting [66]. Majhail et al. included only patients with ferritin levels of >1000 ng/mL and LIC of ≥5 mg/g on liver R2 MRI in a prospective study of iron overload management in 147 adult allo-HSCT survivors, and 16 out of 147 patients had significant iron overload. Based on physician and patient preference the patients were divided into 3 different treatment modality groups: 5 of the patients were followed by observation only, 8 patients had phlebotomy, and 3 patients were treated by deferasirox. Deferasirox decreased the LIC after 6 months of therapy in all 3 patients. The authors concluded that phlebotomy and deferasirox appeared to be effective alternative treatments of iron overload in post allo-HSCT [69]. A phase IV open-label study showed a significant reduction in serum ferritin and LIC over 1 year in allo-HSCT recipients treated with deferasirox [70]. In a recent study of 76 nonthalassemic patients, the authors reported a deferasirox-induced negative iron balance in 84% of patients after initiating it at a median of 168 days after HSCT. The drug-related adverse events were increased blood creatinine (26%), nausea (9%), and abdominal discomfort (8%) [71].

Deferasirox has also been tried during the administration of conditioning regimens and it was found to be safe and reduced the appearance of labile plasma iron shortly after allo-HSCT in a preliminary study [72]. The studies of deferasirox in post-HSCT survivors with iron overload are summarized in Table 3. Visani et al. evaluated the effect of deferasirox on the restoration of normal hematopoiesis in 8 HSCT recipients and all patients experienced an increase in hemoglobin levels with a reduction of transfusions, followed by transfusion independence. This interesting result shows us that deferasirox might have a beneficial effect on hematopoietic recovery after allo-HSCT [73].

In conclusion, iron overload is a common complication and this possibility should be considered in all HSCT recipients. Patients will benefit from careful screening and diagnostic tools such as serum ferritin and transferrin saturation levels and LIC by MRI or biopsy. The initiation of phlebotomy and/or iron chelation therapy if needed will prevent patients from end-organ toxicities. Further studies should be conducted in order to determine better preventive measures and to avoid iron overload, as well as to improve survival in HSCT settings.

## Figures and Tables

**Table 1:**
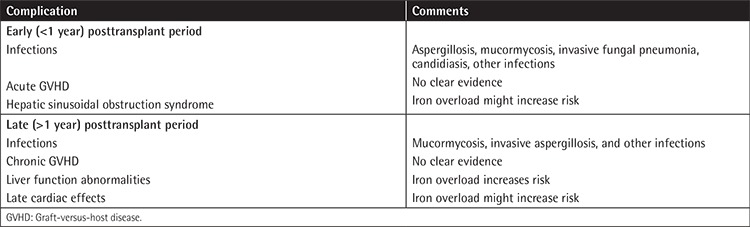
Iron overload-related complications after hematopoietic stem cell transplantation (adapted from Majhail et al. [10]).

**Table 2:**
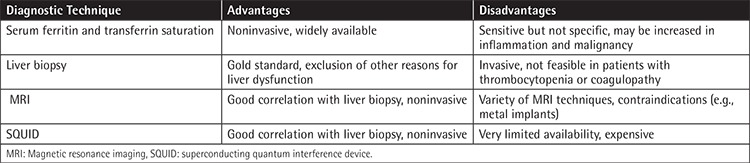
Diagnostic techniques for determining iron stores.

**Table 3:**
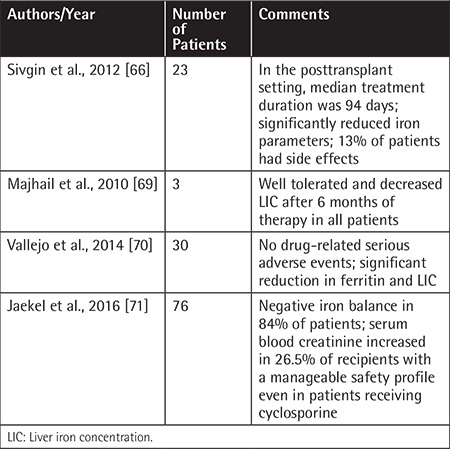
Management of iron overload with deferasirox in hematopoietic stem cell transplantation recipients.
